# Gradient Microstructure in a Gear Steel Produced by Pressurized Gas Nitriding

**DOI:** 10.3390/ma12223797

**Published:** 2019-11-19

**Authors:** Ran Yang, Guilin Wu, Xiaodan Zhang, Wantang Fu, Niels Hansen, Xiaoxu Huang

**Affiliations:** 1 International Joint Laboratory for Light Alloys (MOE), College of Materials Science and Engineering, Chongqing University, Chongqing 400044, China; ranyang@cqu.edu.cn (R.Y.);; 2 Shenyang National Laboratory for Materials Science, Chongqing University, Chongqing 400044, China; 3 Department of Mechanical Engineering, Technical University of Denmark, DK-2800 Lyngby, Denmark; 4 State Key Laboratory of Metastable Materials Science and Technology, College of Materials Science and Engineering, Yanshan University, Qinhuangdao 066004, China; 5 Technical University of Denmark, Risoe Campus, DK-4000 Roskilde, Denmark

**Keywords:** gear steel, pressurized gas nitriding, microhardness, gradient microstructure

## Abstract

A tempered martensitic gear steel (18CrNiMo7-6) sample was nitrided on two sides using a 5 atm pressurized gas at 530 °C for five hours. The nitrided sample was characterized by means of microhardness, X-ray diffraction, scanning electron microscopy, and transmission electron microscopy. A microhardness gradient was identified over a distance of 1000 µm with hardness values from 900 HV0.1 at the surface to 300 HV0.1 in the center matrix. The gradient microstructure was mainly divided into three zones: (i) a nitride compound layer at the top surface (~20 µm thick), (ii) a diffusion zone with containing precipitates (~350 µm thick), and (iii) the center matrix of the tempered martensite. Compared with carburized sample, the harder surface of the nitrided one ensures a better performance of the present pressured gas nitrided gears.

## 1. Introduction

Wind energy is an important source of electrical power. The efficiency of wind turbines can be improved by having gears with better performance, e.g., an increased life time. Increasing the life time of gears requires precise dimensions and a better surface finish of the gears [1,2,3,4,5,6]. To delay failure, a hard surface layer and tough interior of the same gear are generally required [7,8]. Therefore, surface hardening treatments are usually used to harden the surface while leaving the interior untreated. Examples of such surface hardening processes widely used are nitriding [9,10], carburizing [11,12] and shot peening [13,14].

Gas nitriding is used as a surface hardening treatment because it is performed at a relatively low temperature, which limits the size change and dimensional distortions of components during nitriding [15,16,17,18,19]. However, this process normally takes a long time, from dozens of hours to a week. However, the nitriding process can be greatly accelerated by using a high-pressured gas, because high pressure can both suppress the decomposition of ammonia and enhance the nitriding potential and kinetic energy of the gas in nitriding furnaces [20,21]. A pressurized gas nitriding (PGN) treatment is normally operated under a high pressure up to 6 atm. Keeping other nitriding conditions the same, the higher the pressure, the thicker the case-depth of nitrided layers. For a sample nitrided by PGN under 6 atm, the efficiency is nearly 10 times higher than that for conventional nitriding under atmospheric pressure [22,23,24]. 

Usually, the nitrided surface region is subdivided into a compound layer adjacent to the surface, mainly composed of iron nitrides, and a diffusion zone, where, at the nitriding temperature, the nitrogen is either dissolved in the octahedral interstitial sites of the body-centered cubic Fe parent lattice (carbon steels) or precipitated as alloying element nitrides (alloyed steels) [17,18,19,25]. The compound layer is beneficial for resistance against wear and corrosion. The diffusion zone can increase the fatigue resistance of gears; wear resistance can also be enhanced if the alloying element nitrides are precipitated in the diffusion zone [23,24]. Up to now, many studies have reported on the gas nitriding process. However, microstructures with a high resolution along the case depth after nitriding process are not well understood.

In a previous work, we reported on the gradient microhardness of a PGN gear steel [26], which is typically carburized. In the present experiment, the microstructures of the nitrided gear steel are characterized using transmission electron microscopy (TEM) at several specific areas from the surface to the center matrix, which has hardly been done in previous investigations, and the relationship between the microhardness and the microstructures from the surface to the center matrix are discussed.

## 2. Experimental Procedure

18CrNiMo7-6, a typical gear steel, was used in the present work; its chemical composition is listed in Table 1. The base materials were tempered martensitic plates, which were obtained by the following heat treatments: (i) a solid solution treatment at 825 °C for 1 h followed by oil cooling and (ii) tempering at 180 °C for 2 h followed by air cooling. The PGN device employed in this study was a double-layer structure designed to treat samples under higher nitriding pressures by using a pressure-balancing. Details of the furnace can be found in reference [23]. The PGN treatment was carried out at 530 °C for 5 h under flowing NH_3_ and H_2_ with a nitriding potential of r_N_ = 0.26 atm^−1/2^ (NH_3_ flux: 0.2 L/min) under a gas pressure of 5 atm on both sides. The dimensions of the samples for nitriding were 110 mm long, 13 mm wide, and ~1 mm thick. Before nitriding, the sample surfaces were prepared by mechanical grinding using silicon carbide abrasive papers under flowing water. 

The microstructures of the as-received and nitrided steel were characterized by backscatter electron (BSE) imaging and electron backscattering diffraction (EBSD) with a Zeiss (Oberkochen, Germany) Supra 55 scanning electron microscope (SEM) and TEM with a JEOL (Akishima, Tokyo, Japan) JEM-2100 electron microscope. Specimens for EBSD mapping were prepared by mechanical polishing down to colloidal SiO_2_, and samples for BSE observation were slightly etched by 3% nital after mechanical polishing. TEM specimens near the sample surface were prepared by a focused ion beam (FIB) with a Zeiss Auriga Dual beam station. The other TEM samples were prepared by mechanical grinding and thinning by twin-jet polishing in an electrolyte consisting of 10 vol.% perchloric acid and 90 vol.% acetic acid at −20 °C. The precipitate/particle size was measured by using the Image-Pro software by drawing lines along precipitates or particle boundaries then calculating the mean Feret diameters. The phase composition of the nitrided surface layer, diffusion zone, and the center matrix were examined by X-ray diffraction (XRD) using Cu-K_α_ radiation (λ = 1.5406 Å) operated at 40 kV and 40 mA on planes parallel to the nitrided surface (110 × 13 mm). XRD patterns were recorded with a step size of 0.01° and a step duration of 1 s over an angular range of 30°–90°. The penetration depth according to the set-up was estimated to be about several micrometers.

The microhardness profile as a function of the distance of the nitride sample was measured over the whole cross-section using a Vickers hardness tester with a load of 100 g and a dwell time of 10 s. The microhardness values of each depth were obtained from the average of at least 8 measurements. The case depth was determined according to the procedure by measuring the distance from the surface to the point where the hardness was 50 HV higher than that of the center matrix [27].

## 3. Results

### 3.1. Initial State

**Figure 1:**
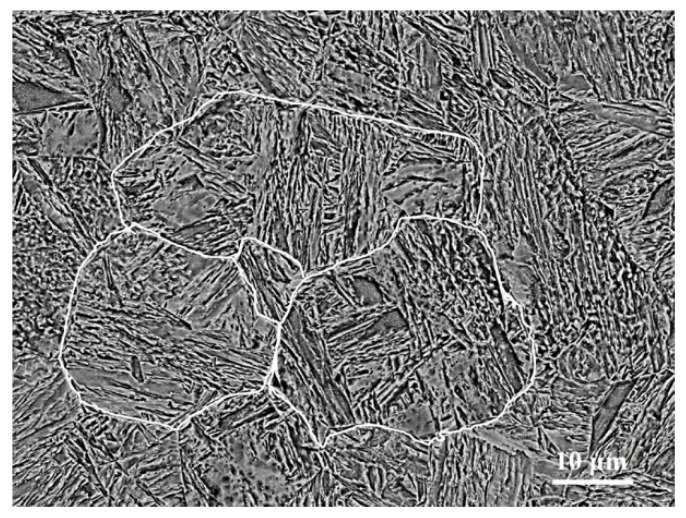
SEM-BSE (scanning electron microscope-backscatter electron) image of the tempered martensite prior to nitriding. The white lines mark the original austenite boundaries.

Figure 1 shows the BSE image of the tempered sample prior to nitriding. The microstructure was a typical lath martensite in low carbon steels, in which several packets could be seen in one original austenite grain and parallel blocks could be seen in each packet [28]. The hardness of the tempered martensite microstructure prior to nitriding was 442 HV0.1.

### 3.2. Hardness Gradient

**Figure 2:**
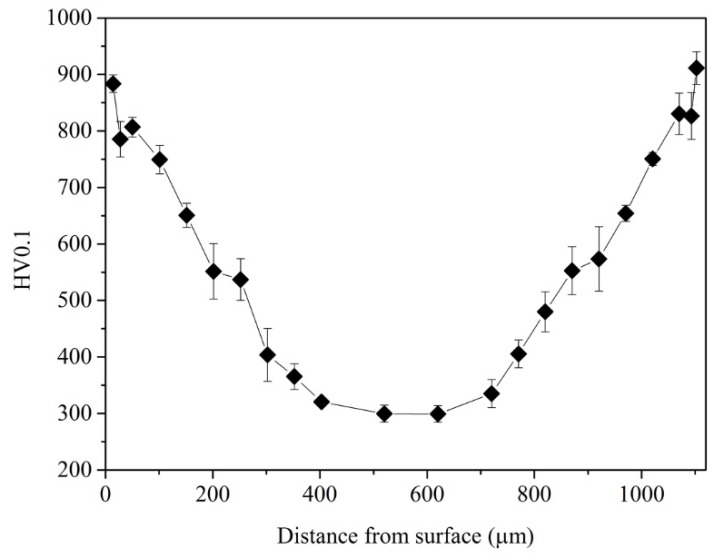
Variation of Vickers microhardness as a function of the distance from the surface. The hardness changed similarly along the directions from two surfaces.

Figure 2 shows the Vickers microhardness profile from the two surfaces to the center of the nitrided sample. It can be seen that the hardness changes from the two surfaces to the center were comparable. A maximum hardness (900 HV0.1) near the top surface was observed corresponding to a microhardness increase of about 600 HV0.1 or an enhancement of three times compared with the center matrix (300 HV0.1). The case depth was found to be about 370 µm. Note that the center hardness was lower than that prior to nitriding (442 HV0.1). 

### 3.3. XRD Results

**Figure 3:**
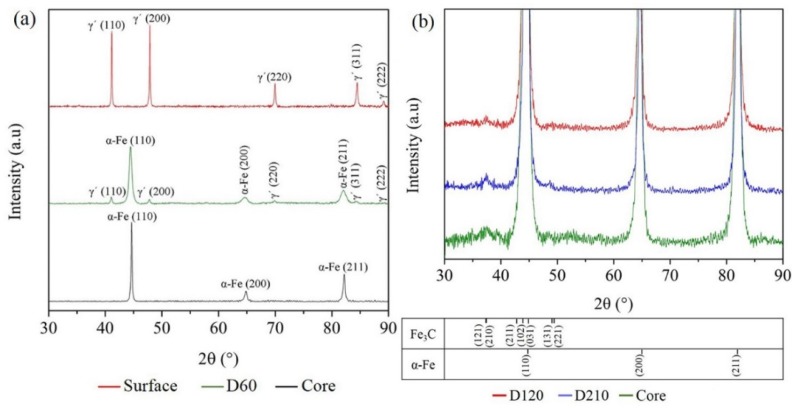
(**a**) XRD patterns taken from the surface, 60 µm from surface, and in the core of the nitrided sample. Only one series of diffraction peaks can be seen from the surface data, corresponding to the γ´-Fe_4_N iron nitride phase. The diffraction patterns of the core correspond to α-Fe (tempered martensite). At the depth of about 60 µm below the surface, diffraction peaks both from γ´-Fe_4_N and α-Fe can be identified. (**b**) Magnification of XRD patterns with range of the lower intensity of 120 µm, 210 µm, and the core center. They are composed of α-Fe and Fe_3_C.

XRD analyses were done at several depths of the nitrided steel from the top surface to the center matrix. As shown in Figure 3a, at the top surface only one series of X-ray diffraction peaks could be seen, which corresponded to the γ´-Fe_4_N iron nitride phase. This finding shows the formation of a compound layer after nitriding. At a depth of about 60 µm (D60), diffraction peaks from two phases could be seen. These two phases were identified as γ´-Fe_4_N and martensite (α-Fe), illustrating a tempered martensite structure containing precipitates of γ´-Fe_4_N [18] (see the TEM Results section for more details). In the center matrix, only martensite could be seen. Moreover, the diffraction peaks of the α-Fe for D60 were wider than those obtained from the center matrix. This indicates a higher concentration of nitrogen in the iron interstitial sites in this area, and it also indicates that this was a diffusion zone. 

The XRD patterns of nitrided steel at the depth of 120 µm (D120), 210 µm (D210) and the core center are shown in Figure 3b (to clearly show the precipitate peaks, only the low intensity part is shown). It is clear that martensite can be seen in all these XRD patterns. A diffraction peak in D120 corresponding to the (121)/(210) crystallographic planes of cementite can been barely seen. However, more clear diffraction peaks corresponding to cementite can be observed in the D210 and core matrix patterns. These results indicate that the volume fraction of cementite of the nitrided sample increased with the increasing depth from the surface to the center.

### 3.4. SEM Results

**Figure 4:**
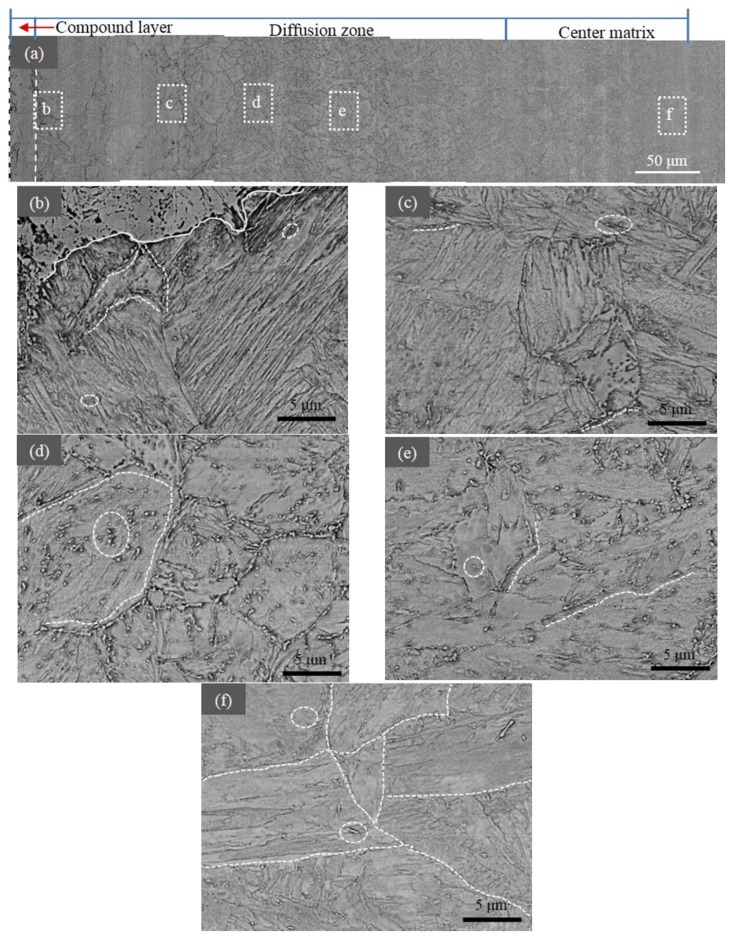
SEM-BSE images of the nitrided specimen at different depths. (**a**) Cross-sectional microstructure. Three zones according to the microstructure are marked, and the thin compound layer is indicated by two parallel white dashed lines. (**b**) The microstructure of the diffusion zone at depth of 30–50 µm. The up side is nearer to the surface. The interface between compound layer and diffusion zone is marked by the white line, and some precipitates are marked by white dashed lines and circles. (**c**) The microstructure of diffusion zone over the depth of 180–200 µm. The presence of precipitate particles is marked using white dashed lines and circles. (**d**) The microstructure of a carbon-rich zone. The presence of precipitate particles is marked using white dashed lines and circles. (**e**) The microstructure of the diffusion zone from 250 to 260 µm. Precipitates are indicated by white dashed lines and circles. (**f**) The microstructure of the center matrix, whose boundaries are marked by white dashed lines and some precipitates, are marked by white dashed circles.

Figure 4 shows the SEM micrographs of the nitrided sample. By using the microstructural features, the microstructure could be mainly divided into three zones. The top zone was the compound layer that extended to a depth of about 20 µm (Figure 4a). The depth and the constituents of this layer were dependent on the nitriding parameters, such as nitriding potential, temperature and time [29]. For the present nitrided sample, only the γ´-Fe_4_N phase was observed in the compound layer, as identified by XRD in Figure 3a. Beneath the compound layer, there was a diffusion zone (Figure 4b–e). The structure was a tempered supersaturated martensite containing dispersed precipitates and is enclosed by circles in the figures (see TEM Results section for more details). In the upper part of this zone (20–200 µm) (Figure 4a–c), lamellar structures can be seen, which are the compounds of alternated lamellae of martensite and γ´-Fe_4_N nitride. At deeper locations (200–240 µm) (Figure 4d), carbon-rich domains were formed. The presence of nitrogen led to the decarburization of the nitrided case and the formation of carbides aligned along boundaries, including both original austenite boundaries and martensite block boundaries [30,31]. The third zone was the center matrix (Figure 4f), which was also composed of tempered martensite with precipitates. However, as seen in Figure 4e there were more precipitates, mostly lying in boundaries compared to the center matrix (Figure 4f). Figure 4f also shows that the microstructure coarsened after nitriding. 

**Figure 5:**
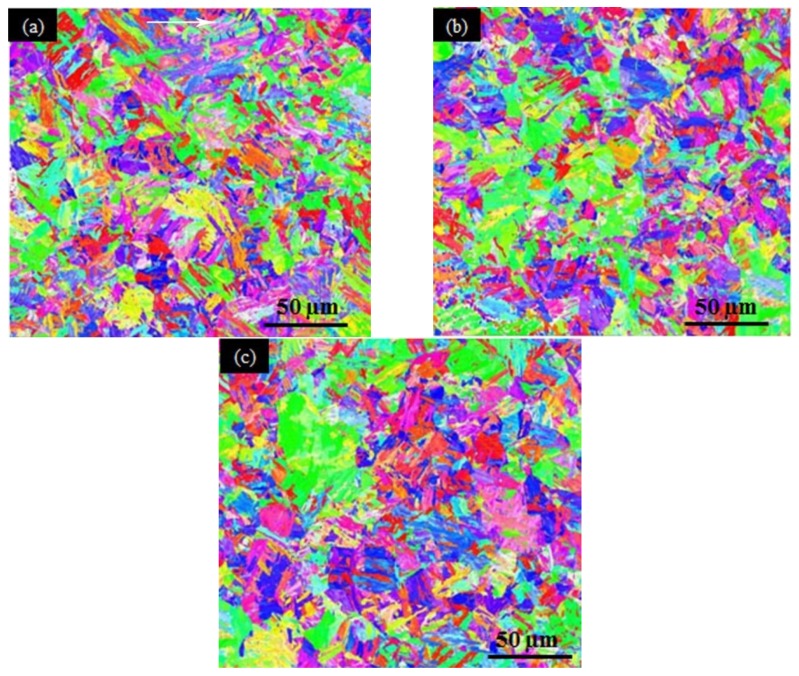
EBSD (electron backscattering diffraction) orientation maps of the nitrided sample at different depths: (**a**) 20 μm–210 μm from the surface; (**b**) 200 μm–390 μm from the surface; (**c**) 390 μm–580 μm from the surface.

EBSD orientation maps of the nitrided sample (Figure 5) were also obtained from the surface to the center. However, only small differences in grain size and crystallographic orientation were found. Nevertheless, they were all composed of tempered martensite without apparent texture.

### 3.5. TEM Results

**Figure 6:**
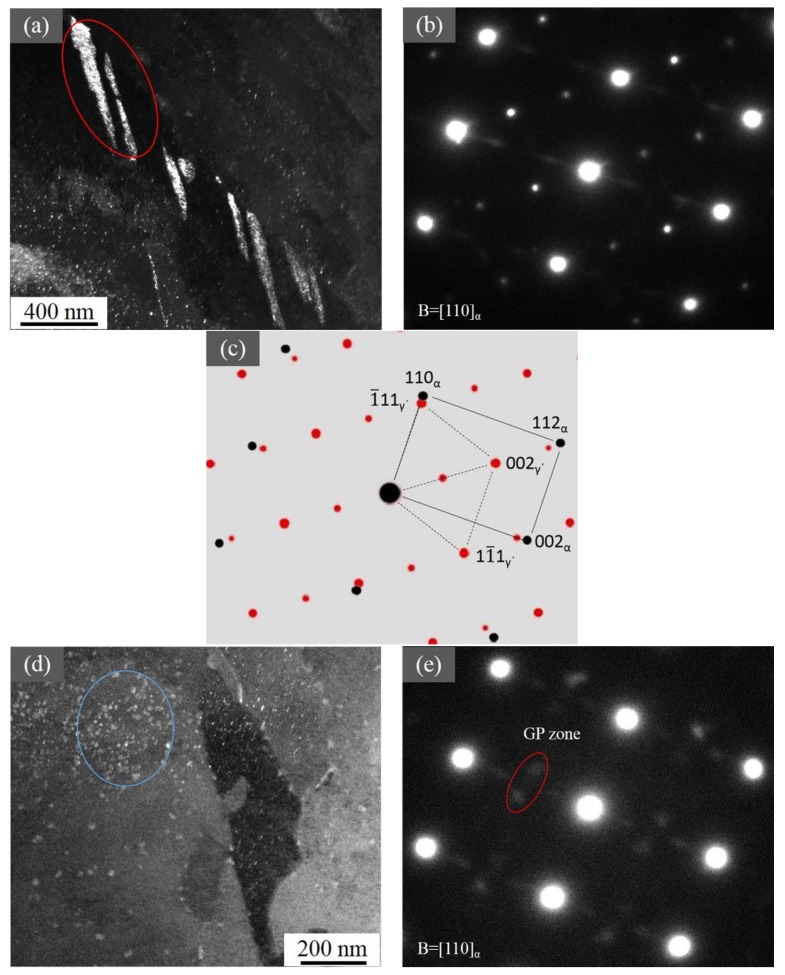
(**a**) TEM image of the nitrided steel at the depth of 40 µm, with Fe_4_N as marked by the red circle, and (**b**) the corresponding SAED (selected area electron diffraction) of Fe_4_N. (**c**) The illustration of (**b**). (**d**) Dark field image of GP zones and CrN as marked by the blue circle and (**e**) the SAED of them.

As precipitates were seen in the SEM images, TEM investigations were performed to reveal the detailed structure and morphology of precipitates in the nitrided steel. At a depth of about 40 µm below the surface, there were precipitates that exhibited a plate-like morphology (a thickness of ~45 nm) with a length around 340 nm in the α-Fe matrix, as shown in Figure 6a. The corresponding selected area electron diffraction (SAED) pattern (see Figure 6b), recorded in the (110)_α-Fe_ zone axis, shows diffraction spots from the α-Fe matrix and Fe_4_N. Its schematic representation in Figure 6c indicates that the Fe_4_N had the following orientation relationship (OR) with the matrix: (110)_α-Fe_ ∥ (110)_γ´_; [110]_α-Fe_ ∥ [111]_γ´_.

Figure 6d shows the TEM image and corresponding SAED along the [011]α-Fe with the superlattice diffraction spots of N-rich Guinier–Preston (GP) zones, both of which fit well with previous results in references [32,33]. These streaks of intensities were a consequence of strain broadening along [100]α-Fe directions with intensity maxima at positions corresponding to the diffraction spots of the CrN of rock-salt crystal structure type oriented in the Baker–Nutting OR ((100)α-Fe ∥ (100)MeN, [010]α-Fe ∥ [011] MeN) with respect to the α-Fe matrix.

**Figure 7:**
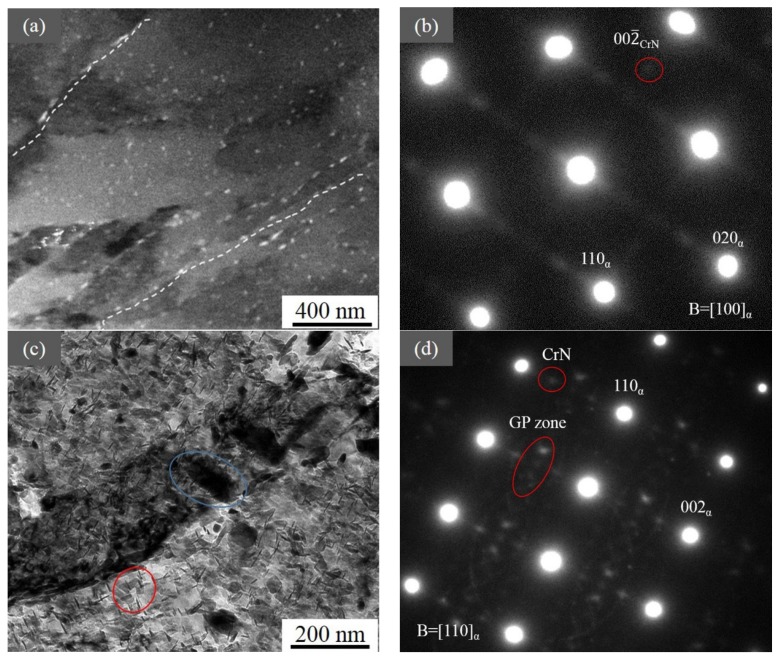
(**a**) TEM image of the nitrided steel at the depth of 120 µm. The white lines indicate the plate-like precipitates along the boundaries, and the corresponding SAED is shown in (**b**). (**c**) TEM image of the nitrided steel at the depth of 210 µm, as well as (**d**) corresponding SAED. In (**c**), the blue circle marks the Fe_3_C, the red one indicates GP zones and CrN, and their diffraction spots are seen in (**d**).

Over the depths of 120 and 210 µm (Figure 7), the precipitates were still N-rich GP and CrN with larger sizes. However, at the depth of 210 µm, the morphology of the precipitates became plate-like [32], and we could see large cementite particles (average diameter: 159 nm) mostly distributing near boundaries. 

**Figure 8:**
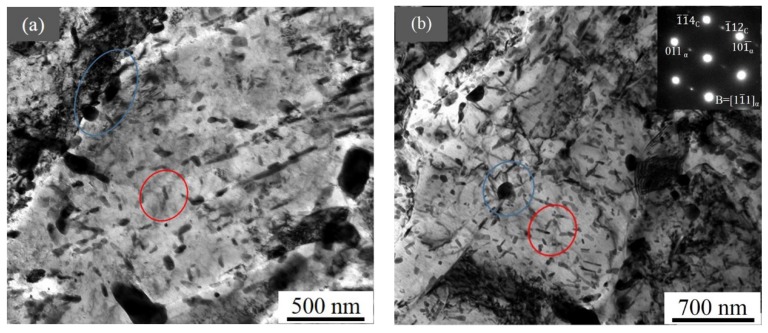
TEM images of the nitrided steel (**a**) at the depth of 360 µm and (**b**) in the center matrix with the SAED inserted. The blue circle shows the large globular Fe_3_C, and the red circle marks the small rod-like one.

At the depth of 360 µm from the surface and further to the center matrix, as shown in Figure 8, there were only two types of particles: large globular ones mostly distributed near boundaries and small rod-like ones present inside the grain (Figure 8a). These particles were identified as cementite according to the SAED (Figure 8b) [34], and the sizes of these cementite particles remained almost stable. The volume fraction of the large globular cementite decreased slightly but increased for the small rod-like cementite with the depth going deeper.

Table 2 summarized the types, sizes and volume fractions of all particles determined by TEM along the depth of nitrided sample.

## 4. Discussions

In the present experiment, gas nitriding was successfully applied to the gear steel, which was demonstrated by the formation of a hard surface layer with a depth of about 370 μm. This layer had a gradient structure, which was analyzed, while the total difference in hardness was 600 HV0.1 from the surface to the center. The surface layer is hard compound layer with a thickness of 20 μm. The diffusion layer had different origins of hardness relating to the formation of precipitates and nitrogen concentration variations [18].

### 4.1. Precipitate Along the Depth

The applied nitriding potential determines the phases formed: a high nitriding potential favors the formation of Fe_4_N, while a low nitriding potential favors the formation of nitrogen-expanded martensite. According to the XRD results, the compound layer was a solid solution composed of Fe_4_N (γ´) and insoluble nitrogen. Moreover, Fe_4_N was contained at least until the depth of 120 μm (Figure 4c). In addition to Fe_4_N, there were fine N-rich GP zones and nano-sized CrN precipitates, which could be observed until the depth of 210 μm. However, the average size of these nano-scale precipitates at the depth of 40 μm was the smallest (~9 nm). At the depth of 120 μm, this size was similar to that at the depth of 210 μm, but the volume fraction was lower. At the same time, above 120 μm, the nano-scale precipitates were mostly equiaxial. Nonetheless, they were plate-like below it. These were attributed to the shortage of nitrogen above 120 μm because of the formation of the Fe_4_N-consuming part of it [35].

For the cementite, the size was almost unchanged along different depths for both large globular and small rod-like particles. However, the volume fraction increased slightly for the large ones along the direction from the center to the surface, and it decreased steeply for the small ones—almost disappearing until 210 μm. The reason for this is related to the decarburization, during which the presence of nitrogen can dissolve small chromium-containing carbides (cementite), because chromium prefers to react with nitrogen over carbon. Therefore, CrN could be identified at the depth of 40 to 210 μm after nitriding. At the same time, the carbon-rich domains were formed below 210 μm.

### 4.2. Influence of Microstructure on Hardness

After nitriding, the microstructures reviewed by EBSD were similar at different areas along the depths of the nitrided sample (see Figure 5). Therefore, the microhardness gradient along the sample depth was not caused by the grain or subgrain structures and/or textures. It is thus believed that the microhardness gradient could be caused by the nitrogen content and precipitates or particles, which are closely related to pressured gas nitriding.

Since the compound layer was composed of Fe_4_N (γ´), which was connected by the covalent bond, it had the highest hardness value.

In the diffusion zone, as shown in Figure 4a, the reduction of hardness could be attributed to the decreasing of nitrogen concentration dissolved in the martensitic matrix and the changing in precipitate density, size and type (Figure 4, Figure 5, Figure 6, Figure 7 and Figure 8, Table 2). In the upper area of diffusion zone, there were Fe_4_N, fine N-rich GP zones, and nano-sized CrN particles. The density of Fe_4_N and nitrogen concentration decreased with the increased depth, thus causing the decreasing of hardness. GP zones and CrN particles with little density variations contributed to the hardness increment a little. However, this could not compensate for the drop in hardness in the upper area of the diffusion zone because the nano-scale precipitates were too small to harden the steel. In the lower area, both the decrease of the density for GP zones, CrN particles, and large spherical Fe_3_C and the reduction of the solution nitrogen atoms contributed to the hardness decreasing in these areas. Though the density of small rod-like Fe_3_C particles increased with the deeper depth in this area, it could not compensate for the drop in hardness of all other particles.

In the center matrix, the microstructure was still un-nitrided martensite, although it was well recovered, which resulted in a decrease of the hardness in center matrix from 442 HV0.1 before nitriding to 300 HV0.1 after the nitriding process. However, the center matrix has enough hardness required for secure performances of gears [36]. 

### 4.3. Case Hardening

For applications of gears, the case-hardening of the 18CrNiMo7-6 gear steel is most commonly achieved by carburization [37,38]. In this work, nitriding by the PGN process was used instead to create a hard case. The results showed a typical nitrided layer structure consisting of a compound layer adjacent the surface and a diffusion zone beneath this layer. The case depth produced in the present 18CrNiMo7-6 gear steel is similar to that obtained in a typical nitrided steel (38CrMoAlA) treated by PGN using the same nitriding equipment and similar nitriding conditions [5]. This shows that PGN can be applied with good results to this commonly used gear steel.

It has also been shown that the surface hardness of the nitrided 18CrNiMo7-6 gear steel is higher than that of the same carburized component (800 HV) after a comparable processing time [38]. Meanwhile, the PGN is operated under much lower temperatures, thus leading to much smaller dimension changes of gears. A better performance in some aspects of PGN gears compared with carburized ones can thus be expected. 

## 5. Conclusions

The formation of a gradient hardness layer, with a depth of 370 μm, was obtained in a gear steel (18CrNiMo7-6) by nitriding under a 5 atm pressure. An analysis of the hardened layer showed a significant structure variation as a function of depth from the hard nitride layer of the compounds at the surface to a center matrix layer of tempered martensite. Different hardening levels that were related to different precipitates/particles could be observed, and three zones were identified: (i) a surface layer with a hardness of about 900 HV0.1, which was a compound layer consisting of a γ´-Fe_4_N phase with a thickness of 20 μm; (ii) a diffusion zone below the compound layer, in which the changing of precipitate or particle type, density and size resulted in a gradual decrease of hardness; and (iii) a center matrix layer, which was tempered at the processing temperature, thus causing a decrease in hardness compared with the martensite sample prior to nitriding. 

## Figures and Tables

**Table 1:** Chemical composition of 18CrNiMo7-6 steel (wt.%).

Element	C	Si	Mn	S	Cr	Ni	Mo	H	Fe
18CrNiMo7-6	0.15–0.21	0.17–0.35	0.50–0.90	≤0.015	1.50–1.80	1.40–1.70	0.25–0.35	≤2.0 ppm	Balanced

**Table 2:** Type, size and volume fraction of precipitates at different depths.

Depth (μm)	Type	Size (nm)	Volume Fraction
40	GP and CrN	9	0.07
	Fe_4_N	45 (aspect ratio 7.6)	0.08
120	GP and CrN	18	0.06
210	GP and CrN	17	0.09
	Fe_3_C	159	0.17
360	Fe_3_C (small)	28 (aspect ratio 3.1)	0.14
	Fe_3_C (large)	161	0.13
500	Fe_3_C (small)	25 (aspect ratio 2.5)	0.21
	Fe_3_C (large)	154	0.10
